# Using short tandem repeat analysis for choriocarcinoma diagnosis: a case series

**DOI:** 10.1186/s13000-019-0866-5

**Published:** 2019-08-17

**Authors:** Xiaofei Zhang, Kai Yan, Jianhua Chen, Xing Xie

**Affiliations:** 1 Department of Surgical Pathology, Women’s Hospital, School of Medicine, Zhejiang University, Zhejiang, 310006 People’s Republic of China; 2 Department of Reproductive genomics, Women’s Hospital, School of Medicine, Zhejiang University, Zhejiang, 310006 People’s Republic of China; 3 Department of Gynecologic Oncology, Women’s Hospital, School of Medicine, Zhejiang University, Zhejiang, 310006 People’s Republic of China

**Keywords:** Choriocarcinoma, Gestational choriocarcinoma, Non-gestational choriocarcinoma, Complete hydatidiform mole, Genotyping

## Abstract

**Background:**

Choriocarcinoma is a highly aggressive, malignant trophoblastic neoplasm that can be gestational or non-gestational in origin. Accurate discrimination between these two subtypes, the causative pregnancy type, and the pregnancy-to-treatment interval for gestational choriocarcinoma are vital for clinical management.

**Methods:**

Fifteen choriocarcinomas were genotyped using multiplex fluorescent polymerase chain reaction amplification of 15 short tandem repeat (STR) loci and the amelogenin locus (XY determination). Genotype patterns at each locus from tumoral and maternal tissues were compared, and any prior or concurrent mole/placenta was also compared when available. According to STR results showing the presence or absence of the paternal chromosomal complement, the gestational or non-gestational origin of the tumor and the nature of the causative pregnancy was identified.

**Results:**

Fourteen tumors were gestational. Of these, seven were androgenetic/homozygous XX, and two were androgenetic/heterozygous XX, indicating that the causative pregnancies were molar pregnancies. Among the nine molar pregnancies, five were of the occult type. A menopausal patient developed a tumor from a mole that occurred seven years ago, identified by the genetically identical allele from the tumor and prior mole. One tumor originating from a previous mole was interrupted by term delivery. Two tumors found eight weeks postpartum were identified as originating from a prior occult mole. A pelvic choriocarcinoma was separated from a genetically distinct third trimester intrauterine placenta. Five gestational tumors were biparental: 2 XX, 3 XY. Of three ovarian tumors, two were confirmed gestational (1 androgenetic/homozygous XX; 1 biparental XY), and one was an ovarian tumor (XX) with a complete match of the genotype for all 15 loci, therefore ascertaining its non-gestational origin.

**Conclusion:**

Gestational choriocarcinoma can originate in an androgenetic or biparental manner. The majority are androgenetic/homozygous XX, while a large number of them might be occult molar pregnancies. The origin of ectopic androgenetic choriocarcinoma with concurrent intrauterine placenta might be from either dispermic twin gestation (mole and coexistent nonmolar fetus) or an antecedent molar pregnancy. Choriocarcinoma shortly postpartum might not be associated with the last placenta. STR analysis can be useful in distinguishing gestational choriocarcinoma from non-gestational, as well as the causative pregnancy, and serve as a helpful examination tool for guiding clinical management.

## Background

Choriocarcinoma is a highly aggressive, malignant trophoblastic neoplasm that can occur after pregnancy, which is defined as a gestational choriocarcinoma. When choriocarcinoma develops as a component of a germ cell tumor or is related to a somatic mutation of a poorly differentiated carcinoma, it is called non-gestational choriocarcinoma [1]. Most choriocarcinomas originate from pregnancies, whether abnormal or normal. Complete hydatidiform mole (CHM) is the most common causative pregnancy for the development of choriocarcinoma, accounting for more than 50% of cases, although only 2–3% of hydatidiform moles progress to choriocarcinomas. Approximately 25% are associated with term or preterm gestations, and the remaining 25% of cases follow abortion or tubal pregnancy [2, 3]. Although rare, choriocarcinomas that developed from partial hydatidiform moles have also been documented [4, 5].

Considering the distinct genetic origin, immunogenicity, sensitivity to chemotherapy, and prognosis for gestational and non-gestational choriocarcinoma, it is important to distinguish these two subtypes. The genetic origin of gestational choriocarcinoma is different from non-gestational choriocarcinoma in, as the former has a paternal chromosome complement, while the latter has DNA identical to the patient DNA because it originates from the patient [3]. Genetic origin determines the immunogenicity, which results in greater sensitivity to chemotherapy for gestational compared to non-gestational choriocarcinomas [2]. Therefore, gestational choriocarcinoma has a favorable prognosis when appropriately treated.

For gestational choriocarcinoma patients, the International Federation of Gynecology and Obstetrics (FIGO) staging and scoring system based on prognostic factors determines the treatment strategy and patient prognosis [6]. FIGO prognostic factors include the age of the patient, the type of antecedent pregnancy, the number of interval months from index pregnancy to the diagnosis of gestational trophoblastic neoplasia (GTN), pretreatment with human chorionic gonadotropin (hCG), largest tumor size, site of metastases, number of metastases, and history of previous failed chemotherapy [6]. Among all the prognostic factors, the type of antecedent pregnancy and the time interval from index pregnancy to diagnosis are most difficult to identify, especially for those with multiple pregnancies. However, identification of a causative pregnancy for gestational choriocarcinoma is very important because the prognosis of choriocarcinoma secondary to molar pregnancy is much better than choriocarcinoma secondary to term delivery or non-molar pregnancy abortion [7]. The immediate antecedent pregnancy is often assumed to be the causative agent of a choriocarcinoma and is judged as gestational. However, many occult spontaneous abortions exist clinically, and studies have found that the immediate antecedent or concurrent pregnancy is not always the causative pregnancy [8–11]. Furthermore, it is unreliable to distinguish gestational from non-gestational choriocarcinoma based only on clinical information such as patient age, menstrual status, pregnancy history, and tumor location [10–13]. Molecular genetic techniques, such as restriction fragment length polymorphism analysis and microsatellite (short tandem repeat [STR]) genotyping, have proven to be helpful in distinguishing gestational from non-gestational choriocarcinomas and identifying the causative pregnancy for gestational tumors [9–21].

This study presents the clinicopathological features and STR analysis of a series of 15 choriocarcinoma cases, with discrimination between gestational and non-gestational origin and identification of the causative pregnancy. The goal was to summarize the distinct genetic nature of choriocarcinomas and suggest the use of genetic analysis in clinical practice.

## Materials and methods

### Samples

This study was approved by the institutional review board. The files of the Department of Pathology at the Women’s Hospital, School of Medicine, Zhejiang University were searched for cases of choriocarcinoma occurring from 2014 to 2018. Fifteen cases with tumoral and maternal specimens (decidua, endometrium, myometrium, or fallopian tube) in formalin-fixed, paraffin-embedded tissue blocks sufficient for analysis were identified, including one case (#7) with a prior mole specimen from seven years ago, one case (#6) with an immediate antecedent placenta specimen, and one case (#10) with a concurrent placenta specimen. Each patient’s clinical information, including age, presenting symptoms, gravidity and parity information, surgery type, adjuvant therapy, and follow-up were collected.

### Immunohistochemistry analysis

Immunohistochemical staining was performed on formalin-fixed, paraffin-embedded tissue sections using the Envision™ method. Positive and negative controls were used for each test. The immunohistochemical staining was performed with antibodies against the following markers: CK18, hCG, human placental lactogen (hPL), Mel-CAM, P63 and Ki-67.

### STR analysis

The AmpFLSTR Identifiler Plus PCR Amplification Kit (Applied Biosystems, Foster City, CA) was used for this analysis. For each case, a series of ten formalin-fixed, paraffin-embedded tissue sections were prepared,with the first 4-μm section stained with hematoxylin and eosin (H&E) for identification of well-separated areas of tumoral and maternal tissues. The following nine consecutive, unstained 10-μm slides were prepared for microdissection. Well-circumscribed tumor areas and maternal tissues were circled with a marking pen on the H&E-stained slides to guide microdissection of those tissues. Pinpoint selection areas and careful microdissection are essential for avoiding contamination, for the STR assay is highly sensitive. DNA extraction was performed following established protocols [22]. Polymerase chain reaction amplification of 15 STR loci from 13 different chromosomes (chromosomes 2, 3, 4, 5, 7, 8, 11, 12, 13, 16, 18, 19, 21) and the amelogenin locus (for XY determination) was performed, with thermal cycling conditions and capillary electrophoresis carried out according to the manufacturer’s instructions. Capillary electrophoresis data from both the maternal and villous tissues were analyzed to identify alleles at each locus, according to interpretation of the genotyping results [23]. When previous mole specimens or previous/concurrent placenta were available, the results were analyzed by comparing maternal and tumoral data with previous/concurrent mole or placenta tissues.

## Results

Clinicopathological features of all fifteen patients are provided in Table 1. The patients ranged in age from 10 to 55 years (mean: 32y, median: 31 y). Vaginal bleeding (10 of 15 cases) and lower abdominal pain (4 of 15 cases) were the most common clinical presentations. Serum beta-human chorionic gonadotropin (β-hCG) levels at the time of presentation ranged from 98 to > 800,000 mIU/mL. The median number of previous pregnancies and parities were 3 (range: 0–6) and 1 (range: 0–2).

**Table 1:** Clinicopathologic Features and Genotyping Data in 15 choriocarcinomas

Case N	Age (y)	Clinical Presentation	β-hCG at Presentation (mIU/mL)	gestation history	antecedent gestation	Specimen	Diagnosis	Genotyping	causative pregnancy	Therapy, Disease Status, Follow-up (mo)
1	28	Vaginal bleeding,	2770	G4P1 Term(2 y ago) IA(3y,1y ago)	Missed abortion (2 monthes ago)	Hysterectomy tumor	CC	Gestational Biparental,XX	abortion/ term	TP (4 cycles); +EME-CO (4 cycles);+EP-EMA(3 cycles)+ Hysterectomy; NED (33)
2	32	Vaginal bleeding,	37,382	G3P1 Term(10 y ago) MA(8 y ago)	Missed abortion (4 monthes ago)	Uterine curetting	CC	Gestational, androgenetic, XX	Occult Homozygous CHM	TP (2 cycles) NED (25)
3	32	Vaginal bleeding,	6067	G4P2 Pre tern(13 y ago) Term(12,2y ago)	Missed abortion (14 monthes ago)	Uterine curetting	CC	Gestational Biparental,XY	missed abortion / term	TP (4 cycles); +EME-CO (6 cycles);+ Hysterectomy NED (44)
4	10	lower abdominal, pain; Vaginal bleeding,	297,475	G0P0	no	Ovary, pelvic tumor	CC	Nongestational (tumor matches maternal tissue)	no	BEP(6 cycles) Lost to follow-up(6)
5	55	Vaginal bleeding,	531	G3P1	CHM,(7 y ago)	Uterine curetting + mole	CC	Gestational, androgenetic, XX	antecedent Homozygous CHM	MTX(3 cycles), Hysterectomy, NED(23)
6	34	Vaginal bleeding,	22,586	G2P2 Term(12 y ago)	Term(4 weeks ago)	Uterine curetting + placenta	CC	Gestational Biparental,XY	Term pregnancy	EME-CO (7 cycles) NED (23)
7	37	rising β-HCG	98	G3P2, CHM(7 y ago)	Term(5 y ago)	Uterine curetting	CC	Gestational, androgenetic, XX	Heterzygous CHM	EME-CO (7 cycles) NED (23)
8	37	lower abdominal pain	118	G5P1 Term(10 y ago) SA(12y、5 y ago) IA(13y、2 y ago)	IA(2 y ago)	Right ovary	CC	Gestational, androgenetic XX	Occult Homozygous CHM	EME-CO (7 cycles) NED (29)
9	23	rising β-HCG	160	G2P1	CHM,(9 monthes ago)	Uterine curetting	CC	Gestational, androgenetic, XX	Homozygous CHM	MTX(4 cycles), 5-FU* (4 cycles), EMA-CO (4 cycles) NED (45)
10	21	lower abdominal pain	> 800,000	G0P0	no	Pelvic tumor +Concurrent placenta	CC	Gestational, (biparental XY+ Androgenetic XX)	Occult Homozygous CHM/ twim one CHM	EMA-CO (3 cycles) Termination chemothrapy, NED (51)
11	20	Vaginal bleeding,	18,973	G0P0	no	Right ovary	CC	Gestational, Biparental,XY	Occult SP	EMA-CO (7 cycles) NED (60)
12	35	Vaginal bleeding, + rising β-HCG	14,774	G6P2 DA(10y,10y,12y, 12y ago) Term(5y ago)	Term(8 weeks ago)	Uterine curetting	CC	Gestational, androgenetic, XX	Occult Heterzygous CHM	MTX(3 cycles) + ACTD(5 cycles) NED (10)
13	31	Vaginal bleeding,	> 1000	G1P1	Term(12 weeks ago)	Uterine curetting	CC	Gestational, Biparental,XX	Term pregnancy	EMA (6ycles) + TP (4ycles) NED (8)
14	54	lower abdominal pain	9135	G4P1 IA(32y, 31y,28y,ago) Term(29y ago)	CHM,(2 monthes ago)	Uterine curetting	CC	Gestational, androgenetic, XX	Homozygous CHM	EMA-CO(2 cycles) + TP(2 cycles) + Hysterectomy NED (5)
15	28	Vaginal bleeding	21,154	G6P1 IA(5y, 4y,3y,2y,2y ago)	Term(8 weeks ago)	Uterine curetting	CC	Gestational, androgenetic, XX	Occult Homozygous CHM	MTX (4 cycles) NED (7)

*BEP* bleomycin, etoposide, cisplatin, *CC* Choriocarcinoma, *DA* drug abortion, *EMA* etoposide, methotrexate, actinomycin D, *EMA-CO* etoposide, methotrexate, actinomycin D, cyclophosphamide, vincristine, *IA* induced abortion, *MA* missed abortion, *NED* no evidence of disease, *SA* spontaneous abortion, *TP*, paclitaxel-cisplatin

**Fig. 1:**
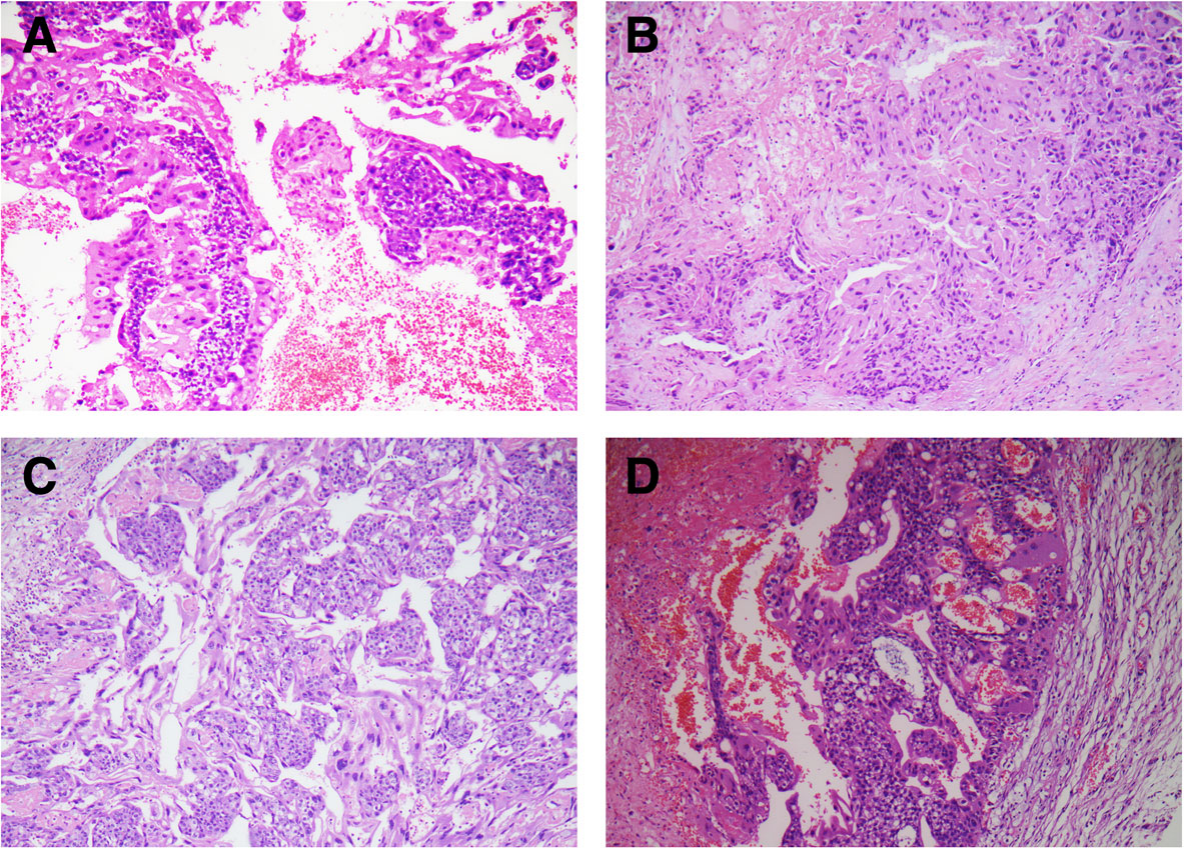
**a** Intrauterine choriocarcinoma (case #7, Fig. 3 contains genotyping data). **b**: Intrauterine choriocarcinoma (case #5, Fig. 4 contains genotyping data). **c**: Pelvic choriocarcinoma with concurrent third trimester placenta (case #10, Fig. 5 contains genotyping data). **d**: Ovarianchoriocarcinoma (case #4, Fig. 6 contains genotyping data)

The diagnosis of choriocarcinoma was made by microscopy evaluation of H&E-stained sections and immunohistochemistry. All tumors were characterized by a mixture of two kinds of trophoblastic cells: mononuclear (including cytotrophoblast and intermediate trophoblast) and syncytiotrophoblast. The typical arrangement pattern of a central core of mononuclear cytotrophoblasts surrounded by a peripheral rim of multinucleated syncytiotrophoblasts could be found (Fig. 1). Chorionic villi could not be seen, except in case #15 with focal, infarct, mature villi eight weeks postpartum.

**Fig. 2:**
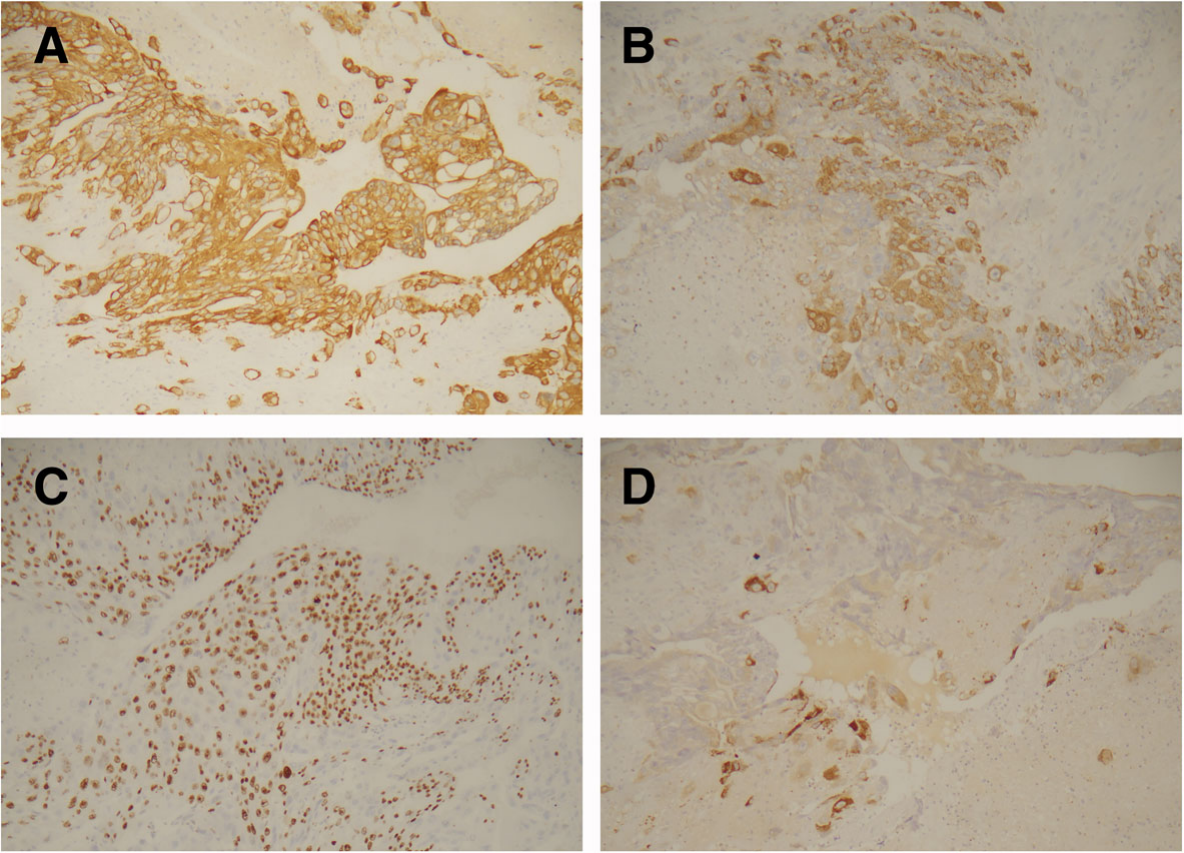
**a** Diffuse, strong, positive staining for CK18. **b**: Syncytiotrophoblast exhibiting diffuse and strong positivity for hCG. **c**: Ki-67 labelling index of 90%.**d**: Intermediate trophoblastic cells expressing hPL

Immunohistochemical analysis found diffuse, strong, positive CK18 staining (Fig. 2a) in all 15 cases. Syncytiotrophoblasts exhibited diffuse and strong positivity for hCG in all 15 choriocarcinomas (Fig. 2b). A high Ki-67 labelling index (> 75%; Fig. 2c) was found in all 15 cases. The intermediate trophoblastic cells expressed Mel-CAM, hPL (Fig. 2d), and P63 in variable proportions.

Eleven cases were uterine tumors, and eight of those were curettage specimens. Four cases were ectopic tumors, with three in the ovary, and one in the pelvis. In case #10, who had an ectopic, pelvic tumor, the uterus contained a 29-week pregnancy placenta with no evidence of intraplacental tumor.

**Fig. 3:**
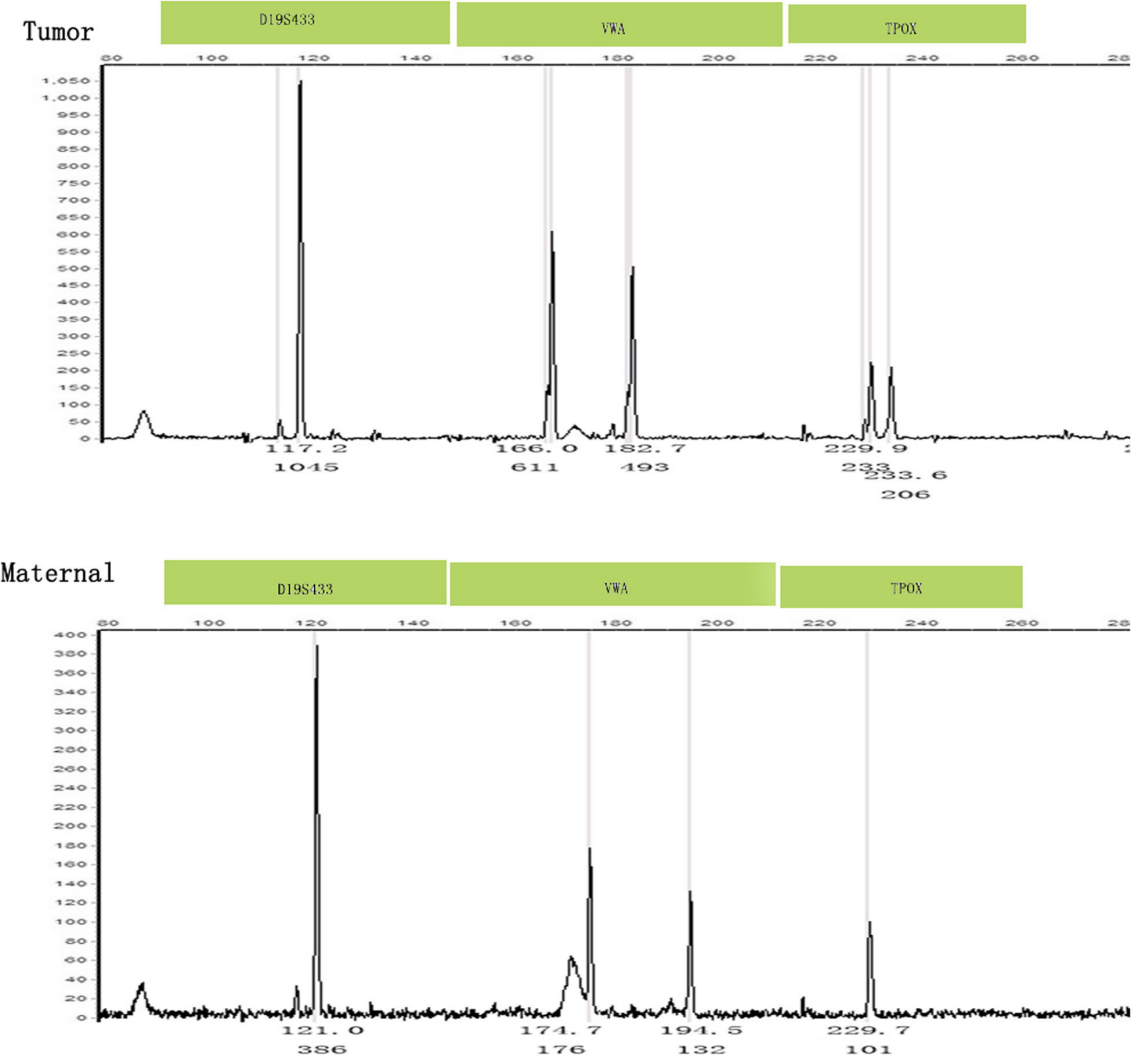
Intrauterine choriocarcinoma (case #7). Genotyping demonstrates that the tumor is purely androgenetic/heterozygous XX with heterozygous alleles VWA that are not present in the maternal sample

**Fig. 4:**
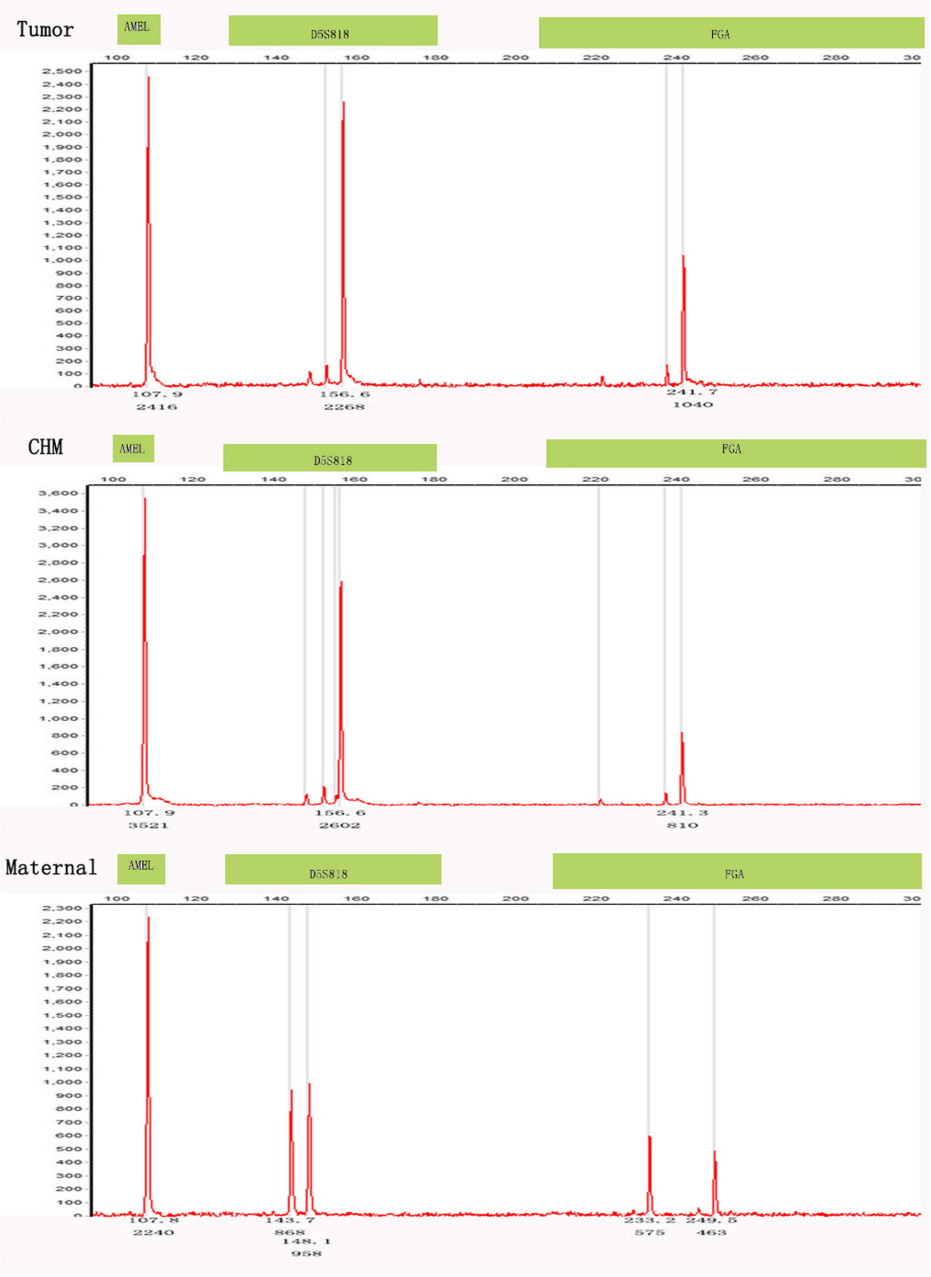
Intrauterine choriocarcinoma (case #5). Genotyping demonstrates that the tumor and the CHM are purely androgenetic/homozygous XX and identical, with different homozygous alleles in D5S818 and FGA compared to the maternal sample

Genotyping results are also provided in Table 1. Fourteen tumors were identified as gestational by STR analysis, nine of which were purely androgenetic with seven homozygous XX and two heterozygous XX (cases #7 and 12; case #7, Fig. 1a for histology and Fig. 3 for genotyping data). Four androgenetic cases (cases #5, 7, 9, and 14) had prior molar pregnancies, of which three were immediate antecedent pregnancies, while case #7 was interrupted by term pregnancy, indicating the clinical assumption that the molar pregnancy was not her immediate antecedent pregnancy. Case #5 was a menopausal patient who developed choriocarcinoma seven years after the prior mole, the genetic analysis of which was performed separately. This confirmed identical DNA patterns as the tumor (case #5; Fig. 1b for histology and Fig. 4 for genotyping data). Since the prior mole specimens were not available for genetic comparative analysis for the remaining three tumors, the genotyping implied but did not confirm that the prior mole pregnancies were most likely the causative factor for the choriocarcinoma. The remaining four cases (#2, 8, 12, and 15) were all genetically homozygous XX, consistent with molar-associated type choriocarcinomas. Case #12 and 15 were identified eight weeks postpartum. The results of genotyping implied that both originated from prior, occult, molar pregnancies, not the antecedent term pregnancies.

**Fig. 5:**
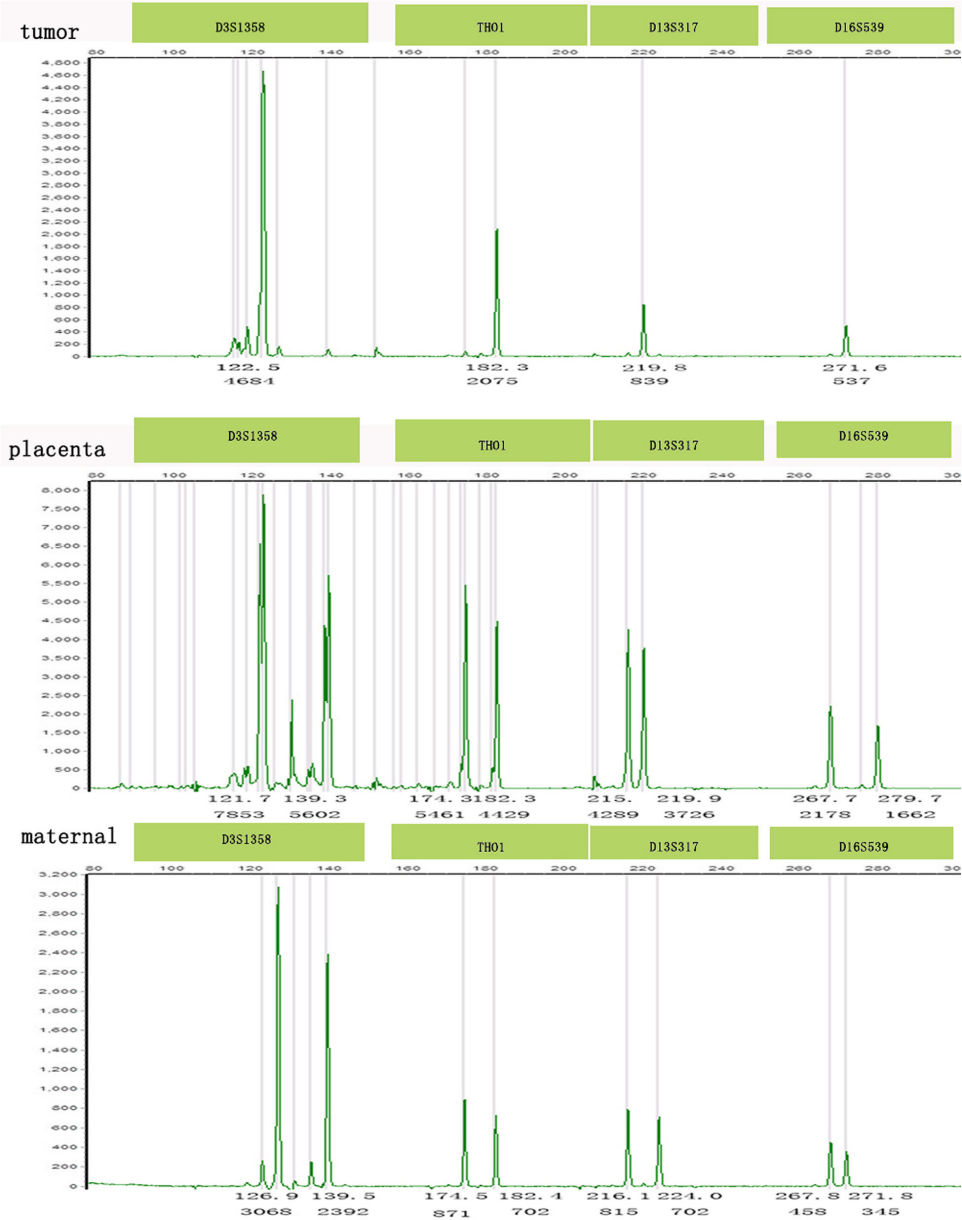
Pelvic choriocarcinoma with concurrent third trimester placenta (case #10). Genotyping demonstratesthat the tumor is purely androgenetic/homozygous XX, with different homozygous alleles in D16S539 compared with the placenta and different D3S1358 and D13S317alleles compared to the maternal sample, indicating that different sperm were involved in the tumor and the placenta

Case #10 presented with a pelvic choriocarcinoma with a concurrent intrauterine live pregnancy. The genotype result of the pelvic tumor was androgenetic/homozygous XX, while the intrauterine, 29-week, gestational placenta was genetically distinct at four loci and biparental XY (D13S317, TPOX, D18S51, and FGA; case #10; Fig. 1c for histology and Fig. 5 for genotyping data), demonstrating that the pelvic tumor did not originate from the placenta. In addition, the placenta was unremarkable on gross and histological examination, excluding the possibility of intraplacental choriocarcinoma. This would be consistent with dispermic gestation: one twin gestation choriocarcinoma is likely originating from a concurrent occult early CHM, coexistent with a nonmolar fetus; the other is a choriocarcinoma originating from a prior occult mole, coexistent with a nonmolar fetus. Considering that there was no identified molar component in the placenta and no known history of a CHM, as this was the patient’s first known pregnancy, it is difficult to identify the origin of the tumor.

Cases #1, 3, 6, 11, and 13 proved to be gestational choriocarcinomas on the basis of their biparental genotyping. These five gestational choriocarcinomas were likely secondary to the formation of a diploid embryo following fertilization of a single ova and sperm. Case #6 presented with uterine choriocarcinomas four weeks postpartum, and the STR results of the last placenta and the tumor were identical, confirming that the tumor originated from the last placenta villi. Cases #1, 3, and 13 were all uterine choriocarcinomas. Both cases#1 and 3 had multiple pregnancies with missed abortions as the immediate antecedent pregnancy. Case #13 (XX) was identified 12-weeks postpartum. Although this was her first pregnancy, we were unable to genetically identify the responsible gestational event. Case #11 was a 20-year old patient with ovarian choriocarcinoma who had no prior pregnancy history. The ovarian tumor had an XY biparental genotype that confirmed the diagnosis of gestational choriocarcinoma. Although we could confirm gestational choriocarcinoma in the above four cases, the causative pregnancy could not be identified because the prior villi/placentas were not available for genetic analysis.

**Fig. 6:**
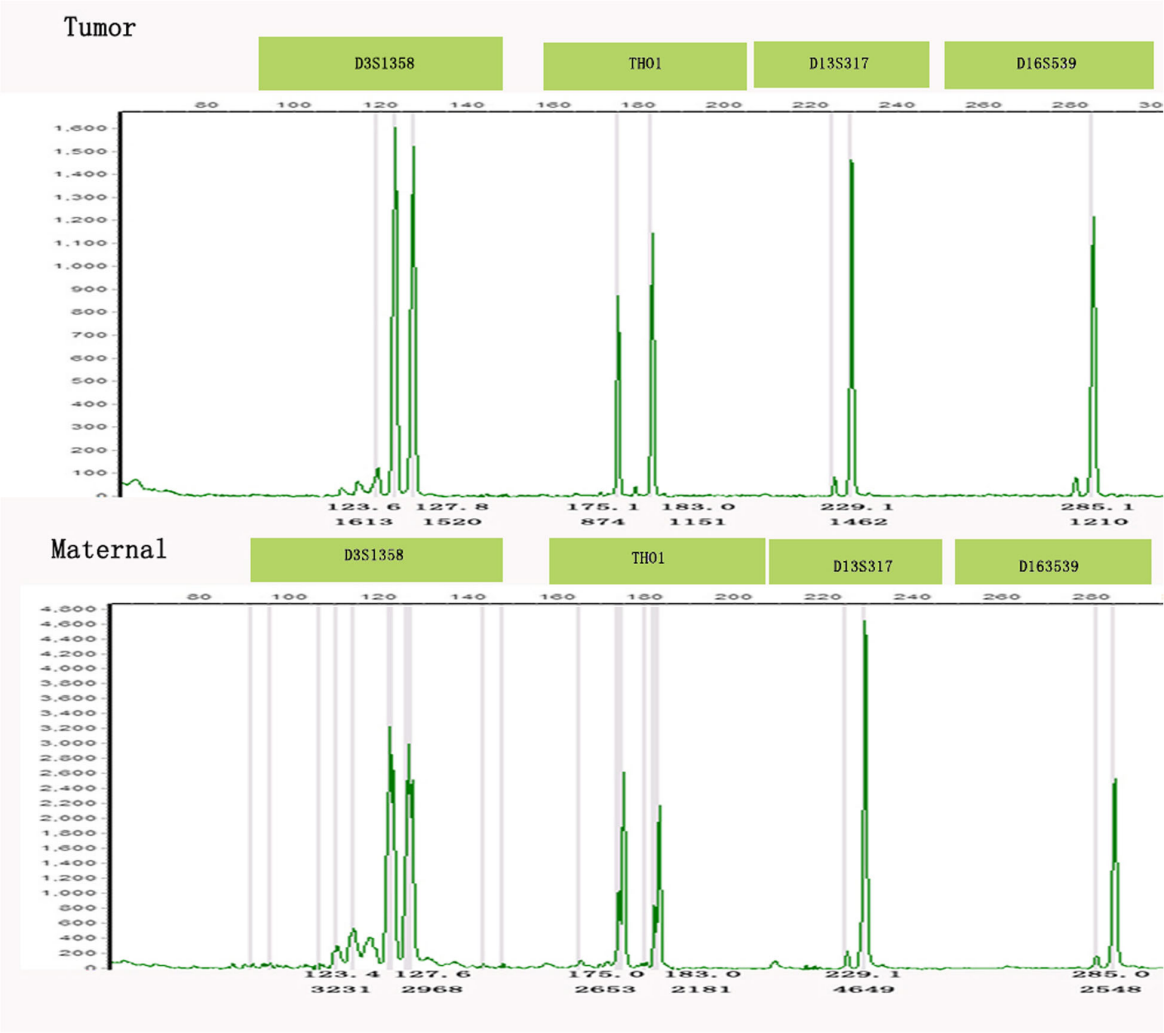
Ovary choriocarcinoma (case #4). Genotyping demonstrates that tumoral and maternal tissues have identical alleles at all loci (D3S1358, THO1, D13S317, and D16S539), indicating a non-gestationalchoriocarcinoma

Case #4 was a ten-year-old girl who was admitted to the hospital for low abdominal pain and vaginal breeding for two weeks. Non-gestational choriocarcinoma was first consideration because of her pre-pubertal age. The result of genotyping was identical alleles in the ovarian tumor and the patient’s normal tissue, which confirmed a non-gestational choriocarcinoma (Fig. 1d for histology and Fig. 6 for genotyping data).

Fifteen patients were divided into three groups according to genotyping results: choriocarcinoma arising from mole, from biparental pregnancy, or non-gestational choriocarcinoma. The follow-up and treatment data were compared between those three groups (Table 2). All nine cases of choriocarcinoma arising from a mole and five cases from a biparental pregnancy were alive with no evidence of disease after at least five months offollow-up. Only one non-gestational choriocarcinoma was lost to follow up. Two of nine patients with choriocarcinoma arising from a mole and two of five from a biparental pregnancy were treated with chemotherapy and hysterectomy. The non-gestational choriocarcinoma patient underwent a right adnexectomy, the remaining patients all received tumor resection or uterine curettage. Three of nine patients with choriocarcinoma arising from a mole were cured with single-agent chemotherapy, four of them with multi-agent chemotherapy, in contrast with three of five patients with choriocarcinoma arising from a biparental pregnancy. Two of nine patients with choriocarcinoma arising from a mole switched to a salvage chemotherapy regimen due to incomplete response to first-line chemotherapy, which was also the case for three cases from a biparental pregnancy. The non-gestational choriocarcinoma patient was treated with BEP (bleomycin, etoposide, and cisplatin).

**Table 2:** Comparison of the treatment and prognosis among choriocarcinoma arising from mole vs from biparental pregnancy vs non-gestational Choriocarcinoma

		choriocarcinoma arising from mole (*n* = 9)	Choriocarcinoma from biparental pregnancy (*n* = 5)	non-gestational choriocarcinoma (*n* = 1)
Surgery (n, %)	Hysterectomy	2 (22%)	2 (40%)	0
	Uterine curetting	5 (56%)	2 (40%)	0
	Pelvic mass resection	1 (11%)	0	1 (100%)
	Adnexectomy	1 (11%)	1 (420%)	1 (100%)
Prognosis(n)	Alive, no evidence of disease	9	5	
	Lost of follow-up			1
Chemotherapy (n, %)	First-line single-agent alone	3 (33.3%)	0	0
	First-line multi-agent alone	4 (44.4%)	2 (40%)	1 (100%)
	Salvage multi-agent chemotherapy	2 (22.2%)	3 (60%)	0

## Discussion

Choriocarcinoma is a highly aggressive, malignant trophoblastic tumor with two subgroups: gestational and non-gestational. Non-gestational choriocarcinomas may arise as a germ cell tumor, most commonly as a component of a mixed germ cell tumor, or as somatic carcinomas, generally as a component of a poorly differentiated carcinoma or adenocarcinoma. Gestational and non-gestational choriocarcinoma differ in genetic origin, immunogenicity, sensitivity to chemotherapy, and prognosis, although they share similar pathological and morphological features. Genetically, gestational choriocarcinoma is considered to be an allograft, is more immunogenic, and responds well to chemotherapy, in contrast to non-gestational choriocarcinomas originating entirely from the patient. Gestational choriocarcinoma has a better prognosis than non-gestational choriocarcinoma [24]. Chemotherapy is the main treatment option for gestational choriocarcinoma, while non-gestational choriocarcinoma is treated with surgery combined with chemotherapy as determined by disease stage. Moreover, the prognosis for gestational choriocarcinoma secondary to molar pregnancy is much better than those secondary to term or nonmolar abortion [7]. Accurate subclassification of the tumor origin, identification of causative pregnancy, and determining the accurate time interval from the index pregnancy to diagnosis are vital for guiding patient management. A number of studies have reported that genetic analysis, especially STR genotyping, may help solve these problems [9–21], but most were case reports, and few were case series. The current study is the second largest case series of the genetic analyses of choriocarcinoma.

Similar to previous reports, our study confirms prior opinion and summarizes the following. In gestational choriocarcinoma, an antecedent pregnancy is not always the causative pregnancy based on STR analysis, which has been proven by numerous similar studies [9, 11, 25, 26]. Since more than 50% of gestational choriocarcinomas followed molar pregnancies, studies reported that molar pregnancy could be the causative pregnancy for the development of choriocarcinoma following the birth of normal children [8, 10, 11, 25]. We assume that the causative pregnancy is most likely a previous mole when the mole is documented in the pregnancy history, whether the immediate antecedent or not [8, 10, 11, 25]. In rare cases, however, this might not be the case. Zhao reported an intrauterine choriocarcinoma patient whose causative pregnancy was a neglected biparental pregnancy, not the only known previous molar pregnancy [10]. In the largest case series, of nineteen gestational choriocarcinoma cases, fourteen were purely androgenetic in origin, identified by STR analysis, while only six of them had a concurrent or prior genetically related CHM [8]. Zhao also identified the causative pregnancies as androgenetic CHM in six of eight gestational choriocarcinomas, with only one case having a prior mole history, but not the immediate antecedent type [11]. Among the fourteen gestational choriocarcinomas in our study, nine were genetically confirmed as androgenetic CHM. Similarly, only four had known histories of a previous mole, but one of them was the antecedent of the choriocarcinoma. Along with previous observations, our study demonstrates the necessity and importance of genetic examinations of choriocarcinoma, since clinical data alone are often unable to accurately identify the causative pregnancy. There may be some occult pregnancies, especially molar pregnancies in the clinic, since histopathological examination of every abortion is not routine, and clinics may fail to distinguish early CHM from abortion. In addition, we also speculate molar pregnancy, as the genetically causative pregnancy of gestational choriocarcinoma, might constitute a higher percentage than previously thought [2, 3], which warrants reviewing a larger number of cases.

Gestational choriocarcinoma can be androgenetic or biparental in origin. The majority were androgenetic/homozygous XX, with the minority being androgenetic/heterozygous XX or XY. The 14 androgenetic cases in Savage’s study were all homozygous XX, while our results showed two of the nine androgenetic were heterozygous XX, which was also found in Bynum’s study [27]. Biparental origin includes full term, non-molar miscarriage, and ectopic pregnancy. The interval time to the development of choriocarcinoma is usually not more than one year after the antecedent pregnancy, whether molar or non-molar. Nevertheless, there still can be a significant time interval between the index molar pregnancy and choriocarcinoma. A few reports found delayed choriocarcinoma originating from a prior molar pregnancy [8, 9, 25, 26]. We observed this in a postmenopausal patient, with an interval of seven years. Another patient, case #7, developed gestational choriocarcinoma with an interval of seven years between the molar pregnancy and choriocarcinoma, interrupted by a full-term pregnancy.

Clinically, intrauterine choriocarcinoma is always assumed to be gestational, except in older women with trophoblastic differentiation within an endometrial carcinoma or choriocarcinoma arising from germ cells. Fallopian tube tumors are also usually gestational [8], while those in other sites (ovary, pelvis) can be non-gestational [3]. It is impossible to ascertain the type of choriocarcinoma based on clinical information alone, such as age, previous pregnancy history, image diagnosis, and histologic features. Genotyping is useful to accurately identify whether an ovarian choriocarcinoma is gestational or non-gestational [11, 12, 14, 16, 18, 19]. In our study, case #4 was a ten-year-old girl, and the tumor was pure ovarian choriocarcinoma histologically, lacking other germ cell components. A non-gestational choriocarcinoma was assumed based on this information, which was confirmed by STR genotype, with the tumor sharing identical STR alleles as the patient. Both case #8 and 11 were ovarian choriocarcinomas of reproductive age. Since case #11 denied having previous pregnancies, gestational or non-gestational origin was difficult to ascertain from the clinical data. We characterized both of these cases as gestational choriocarcinoma by STR analysis with an XY biparental genotype in case #11 and a homozygous XX in case #8.

Choriocarcinomas concurrent with pregnancy are very rare, and most of them are intraplacental choriocarcinomas (IC), defined as choriocarcinomas located within the placenta. IC might be more prevalence than previously thought, as histological examination of the postpartum placenta is not routinely performed, and approximately half of IC cases were asymptomatic [28]. Considering the presence of pregnancy and the observation that some IC cases histologically appear to arise from placental villi [29], choriocarcinoma coexisting with pregnancy is assumed to originate from the current placenta. Among six reported IC cases with STR results, five IC genotypes matched the placental tissue, indicating the IC originated from the concurrent pregnancy [8, 30, 31]. Although recent reports indicate that the current pregnancy may most often be the causative factor, choriocarcinomas concurrent with pregnancy may still arise from previous pregnancies. Yamamoto reported one case that found mismatched STR alleles between the tumor and concurrent villous tissue, suggesting that IC may have arisen from a previous pregnancy [32]. For ectopic choriocarcinoma, we cannot assume that the tumor developed from the current pregnancy or metastatic lesions from the placenta. Savage’s study showed that analysis of any available villous/placental tissue from a concurrent or prior gestational event can determine the causative pregnancy by assessing the genetic relationship between the tumor and the villous tissue [8]. Aranake-Chrisinger presented the first report of a choriocarcinoma originating from a previous occult mole coexisting with pregnancy [9]. Savage’s study and our case #10 had similar findings. A previous molar pregnancy or a twin pregnancy with one complete hydatidiform mole and another normal biparental pregnancy could lead to this discordant genotype between the fetus/placenta and the tumor. Pelvic gestational choriocarcinoma coexisting with an intrauterine pregnancy was the first known pregnancy in case #10, with the results of STR analysis indicating that the tumor was an androgenetic mole, while the placenta was XY biparental. Given the fact that no tumor was found in the placenta during gross or histologic examination, it is challenging to distinguish a previous occult mole from a twin pregnancy as the causative pregnancy without the specimen of the previous mole.

## Conclusion

Gestational choriocarcinoma can have androgenetic or biparental origin, but the majority are androgenetic/homozygous XX, and a large number of them might be clinically occult molar pregnancies. The origin of ectopic androgenetic choriocarcinoma with concurrent intrauterine placenta might be from either dispermic twin gestation (mole and coexistent nonmolar fetus) or an antecedent molar pregnancy. Choriocarcinoma shortly after postpartum might not be associated with the last placenta. STR analysis can be useful for distinguishing gestational choriocarcinoma from non-gestational types, as well as identifying the causative pregnancy, and serve as a helpful examination for guiding clinical management.

## Data Availability

Due to privacy, data are available on request.

## Abbreviations

BEP: Bleomycin, etoposide, cisplatin
CHM: Complete hydatidiform mole
FIGO: International Federation of Gynecology and Obstetrics
GTN: Gestational trophoblastic neoplasia
H&E: Hematoxylin and eosin
hCG: Human chorionic gonadotropin
hPL: Human placental lactogen
STR: Short tandem repeat
β-hCG: beta-human chorionic gonadotropin
